# microRNAs: key triggers of neuronal cell fate

**DOI:** 10.3389/fncel.2014.00175

**Published:** 2014-06-25

**Authors:** Karla F. Meza-Sosa, Gustavo Pedraza-Alva, Leonor Pérez-Martínez

**Affiliations:** Laboratorio de Neuroinmunobiología, Departamento de Medicina Molecular y Bioprocesos, Instituto de Biotecnología, Universidad Nacional Autónoma de MéxicoCuernavaca, México

**Keywords:** miRNAs, neuronal differentiation, neuronal cell fate, neural stem cell, neural progenitors, development, central nervous system

## Abstract

Development of the central nervous system (CNS) requires a precisely coordinated series of events. During embryonic development, different intra- and extracellular signals stimulate neural stem cells to become neural progenitors, which eventually irreversibly exit from the cell cycle to begin the first stage of neurogenesis. However, before this event occurs, the self-renewal and proliferative capacities of neural stem cells and neural progenitors must be tightly regulated. Accordingly, the participation of various evolutionary conserved microRNAs is key in distinct central nervous system (CNS) developmental processes of many organisms including human, mouse, chicken, frog, and zebrafish. microRNAs specifically recognize and regulate the expression of target mRNAs by sequence complementarity within the mRNAs 3′ untranslated region and importantly, a single microRNA can have several target mRNAs to regulate a process; likewise, a unique mRNA can be targeted by more than one microRNA. Thus, by regulating different target genes, microRNAs *let-7*, microRNA-124, and microRNA-9 have been shown to promote the differentiation of neural stem cells and neural progenitors into specific neural cell types while microRNA-134, microRNA-25 and microRNA-137 have been characterized as microRNAs that induce the proliferation of neural stem cells and neural progenitors. Here we review the mechanisms of action of these two sets of microRNAs and their functional implications during the transition from neural stem cells and neural progenitors to fully differentiated neurons. The genetic and epigenetic mechanisms that regulate the expression of these microRNAs as well as the role of the recently described natural RNA circles which act as natural microRNA sponges regulating post-transcriptional microRNA expression and function during the early stages of neurogenesis is also discussed.

## Introduction

**Figure 1:**
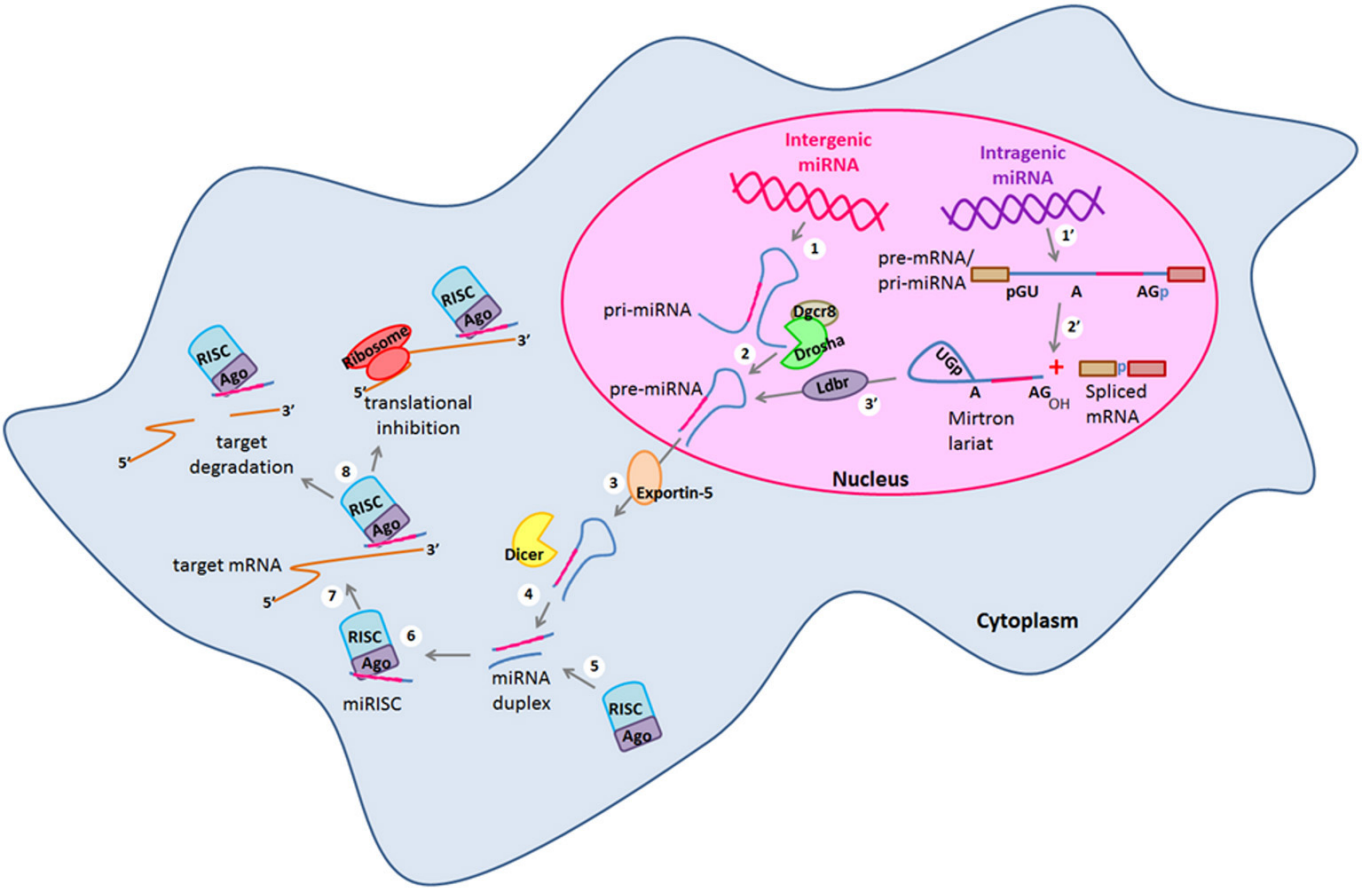
**miRNA canonical and non-canonical biogenesis**. Biogenesis of intergenic microRNAs (miRNAs) begins with the (1) transcription of miRNA genes by the RNA Polymerase II to generate long transcripts known as primary miRNAs (pri-miRNAs) which are then (2) processed by the microprocessor complex formed by Drosha and Dgcr8 in the nucleus and pre-cursor miRNAs (pre-miRNAs) are generated. On the other hand, intragenic miRNAs are also (1′) transcribed as part of the pre-mRNAs of their host protein coding genes which are then (2′) spliced by the alternative splicing machinery giving rise to the spliced mRNA and to a mirtron lariat that contains the future mature miRNA. After that, mirtron lariat is (3′) debranched by the Ldbr enzyme and finally a pre-miRNA is generated. At this point, both canonical and non-canonical pathways take a common course in which (3) pre-miRNAs are transported to the cytosol by exportin-5 to be (4) processed by the type III RNAse Dicer. After that, (5) a miRNA duplex of whom one strand (the mature miRNA) recruits to the RISC complex. (6) The mature miRNA is loaded into the RISC forming the miRISC. (7) miRNA is guided by the RISC to its target mRNA and binds to its 3′ UTR by sequence complementarity. (8) Finally, the mature miRNA negatively regulates the expression of its target genes either by target degradation or by translational inhibition. Dgcr8, DiGeorge syndrome critical region gene 8; Ldbr, lariat debranching enzyme; RISC, RNA-induced silencing complex; UTR, untranslated region.

Embryonic stem cells (ESCs) are characterized by an unlimited self-renewal potential and are pluripotent as they are capable of originate cells of any tissue of the embryo (Gage, 2000; Liu et al., 2009; Xu et al., 2009). As embryonic development progresses, restrictions of cell fate appear and the pluripotent ESCs become multipotent stem-cells which in response to specific stimuli can commit to a given cellular fate even though they still conserve a broad self-renewal potential (Gage, 2000). Hence, these cells are named in accordance to the tissue in which they are found *in vivo*. Neural stem cells (NSCs) and neural progenitors (NPs) are encountered in both embryonic and adult brain (Gage, 2000; Markakis et al., 2004; Li et al., 2008; Liu et al., 2009; Li and Jin, 2010; Roese-Koerner et al., 2013). NSCs and NPs in response to specific intra- and extra-cellular signals can give rise to all the cell types that constitute the central nervous system (CNS) (Reviewed in Gage, 2000; Pérez-Martinez and Charli, 2006; Li and Jin, 2010). NPs divide asymmetrically and one of the two daughter cells acquires a reduced self-renewal potential and eventually irreversibly exits of the cell cycle giving rise to a neuron (Gage, 2000; Li and Jin, 2010). Therefore, for neurogenesis to occur, cellular processes such as proliferation and gene expression regulation must be tightly controlled (Reviewed in Pérez-Martinez and Charli, 2006; Liu et al., 2009; Li and Jin, 2010). In this sense, different transcription factors (TFs) and signaling pathways have been described as crucial players within the intricate gene expression regulatory networks that take place during neurogenesis (Diez del Corral and Storey, 2001; Bertrand et al., 2002; Markakis et al., 2004; Nielsen et al., 2009; Qin et al., 2012). Likewise, epigenetic and gene expression regulation by non-coding RNAs (ncRNAs) have been described as additional and essential regulatory mechanisms for the neurogenic process in which changes in gene expression, protein synthesis and post-translational modifications must be precisely regulated to induce neuronal differentiation and at the same time, maintaining the NSCs and NPs pools (Cao et al., 2006; Li and Zhao, 2008; Liu et al., 2009; Li and Jin, 2010; Meza-Sosa et al., 2012). Within the most studied ncRNAs, microRNAs (miRNAs) have a key role in gene expression regulation at the post-transcriptional level in a wide variety of cellular processes including cell proliferation (Delaloy et al., 2010; Niu et al., 2013), cell fate determination and differentiation (Chen et al., 2004; Makeyev et al., 2007; Li and Jin, 2010; Åkerblom and Jakobsson, 2013), metabolism (Miska et al., 2004; Singh, 2007), and apoptosis (Zhang et al., 2012; Guo et al., 2013) among others. miRNAs are generated from a stepwise process that can be canonical (Drosha/Dgcr8-dependent) or non-canonical (Drosha/Dgcr8 independent) (Figure 1), The canonical biogenesis pathway begins with the transcription of endogenous miRNA genes by the RNA polymerase II giving rise to primary transcripts known as pri-miRNAs which can have a size of hundreds to thousands base pairs (bp) (Bartel et al., 2004). Then, pri-miRNAs are processed in the nucleus where the microprocessor complex conformed by the type III RNAse Drosha and the DiGeorge syndrome critical region gene 8 (Dgcr8) protein cleaves them to originate a miRNA precursor (pre-miRNA) of ~70 bp that by sequence complementarity within itself has a characteristic stem-loop structure (Lee et al., 2002, 2003). pre-miRNAs can also be generated by the non-canonical mirtron pathway that results when a miRNA gene is embedded within the introns of a protein coding gene. Thus, some of the miRNAs generated from these loci are called “mirtrons” (Okamura et al., 2007; Westholm and Lai, 2011). Mirtrons are generated when their host genes are transcribed and then, short introns with potential hairpin enter the mirtron pathway where they are spliced as a lariat in which the 3′ branchpoint is ligated to the 5′ end of the intron then, the lariat debranching enzyme (Ldbr) gives rise to shorter pre-miRNAs that abutted intron-exon boundaries due to their processing by the splicing machinery that can normally continue with the canonical miRNA biogenesis pathway (Okamura et al., 2007; Westholm and Lai, 2011). In this sense, both canonical and non-canonical pre-miRNAs are exported to the cytoplasm by the nucleo-cytoplasmic transport factor Exportin-5 (Yi et al., 2003). Once in the cytosol pre-miRNAs are cleaved by another type III RNAse called Dicer that by leaving the 5′ phosphate and the ~2 bp 3′ overhang characteristic of RNAse III endonucleases, generates an imperfect duplex consisting of the mature miRNA (miRNA-3p or miRNA-5p depending on the case) and its corresponding complementary sequence derived from the other arm of the pre-miRNA (Hutvágner et al., 2001; Du and Zamore, 2005). After that, the strand that will be the mature miRNA (~19–21 bp) (usually the one with the least thermodynamically stable 5′ end in the generated duplex) is selected by the Argonaute (Ago) protein and then loaded onto the RNA-induced silencing complex (RISC) which guides the binding of the miRNA to the miRNA response elements (MREs) that can be found mostly within the 3′ untranslated region (3′ UTR) but also in the 5′ UTR and the coding region of the target mRNAs (Helwak et al., 2013). Importantly, while the canonical MREs in the 3′ UTR are functional when the “seed” sequence of the miRNA (2–8 bp of its 5′ end) is completely paired with the 3′ UTR of target mRNA or with a single mismatch or a non-canonical wobble pairing G:U (Lai, 2002; Lewis et al., 2003); the functionality of the 5′ UTR and coding region MREs mainly depends on a seedless base pairing between the miRNA and the target mRNA (Lal et al., 2009; Helwak et al., 2013).

As mentioned, miRNAs are fine-tuning regulators of gene expression, due to their specific spatio-temporal expression patterns are involved in a wide spectrum of biological processes and thus it is not surprising to find that their expression profiles can be cell- and/or tissue-specific (Smirnova et al., 2005; Hohjoh and Fukushima, 2007; Landgraf et al., 2007; Olsen et al., 2009). Thus, alteration in miRNAs pattern expression results in different diseases (Schratt, 2009; De Smaele et al., 2010; Lau and de Strooper, 2010). Although the participation of miRNAs has been widely documented during the terminal differentiation process of neurons, the role of these post-transcriptional regulators during the first stages of neurogenesis is less understood. Thus, in this review we focus on the role of two subsets of miRNAs (miR-134, miR-137, and miR-25) and (*let-7*, miR-124, and miR-9) that are highly conserved during evolution and play an important role in the early steps of neurogenesis. Moreover, we discuss the possible role of natural occurring circular ncRNAs (natural miRNA sponges) as important regulators of miRNA expression and function during the CNS development. We believe that the information presented in this review would be valuable to potentially develop therapeutic strategies to block or enhance the expression of particular miRNAs and/or their regulators for the treatment of specific CNS pathological conditions.

## miRNAs in the developing CNS

During the CNS development, neurogenesis requires precisely regulated gene expression patterns in which a balance between positive and negative signals must be maintained to generate the correct cell types in the proper time and space (Ivey and Srivastava, 2010; Li and Jin, 2010). As mentioned, miRNAs show cell- or tissue-specific expression profiles (Liu et al., 2009; Ivey and Srivastava, 2010) and thus, they are good candidates to regulate cellular processes in which a very fine-tuning of gene expression is required. In this sense, the first indication of a possible role of small ncRNAs during CNS development came from a study in which Dicer was conditionally knocked-out. These mice present a reduction of forebrain size attributed to an increased apoptosis rate of differentiating neurons (Makeyev et al., 2007). On the other hand, Dicer-deficient NSCs can self-renew but show enlarged nuclei, abnormal differentiation and undergo apoptosis upon mitogens withdrawn suggesting a role of Dicer in NSCs survival and differentiation (Kawase-Koga et al., 2010). Furthermore, Dicer ablation in the cortex and hippocampus, results in microcephaly and in a decreased number of dendrites (Davis et al., 2008). However, as Dicer processes small ncRNAs including short-interfering RNAs (siRNAs) and miRNAs, the defects observed in these Dicer knock-out *in vitro* and *in vivo* systems, could be due to defects in siRNAs and/or miRNAs biogenesis. Direct evidence of the essential role of miRNAs in the CNS development became apparent when abnormal neuronal differentiation and neural tube morphological defects observed in Dicer-deficient zebrafish (Giraldez et al., 2005) were rescued by introduction of miR-430 (Giraldez et al., 2005). In addition, the potential of a single miRNA to function as a cell fate determinant, was demonstrated when overexpression of miR-124 induced a neuronal-like gene expression profile in HeLa cells by targeting non-neuronal genes (Conaco et al., 2006). Moreover, co-expression of miR-124 and miR-9 shifts the cell fate of NPs toward the neuronal fate (Krichevsky and Sonntag, 2006). The importance of miRNAs in CNS development is further highlighted by their interspecies sequence and function conservation (Zhao and Srivastava, 2007; Coolen and Bally-Cuif, 2009; Yuva-Aydemir et al., 2011). Besides, it is important to point out that the identification of particular miRNAs acting as gene expression regulators during the different stages of neurogenesis has been crucial for the better understanding of the CNS development. Particularly, recent studies have identified miR-134, miR-137, and miR-25 as important regulators of NSCs and NPs functions during neurogenesis as described below.

## miRNAs controlling NSCs and NPs proliferation

### miR-134

miR-134 belongs to the miR-379-410 cluster (Rago et al., 2014) and itself is a powerful inducer of pluripotent ESCs differentiation (Gaughwin et al., 2011). miR-134 expression increases in mouse ESCs treated with retinoic acid (RA), favoring ESCs differentiation into ectodermal lineages including neural cells by directly regulating the expression of the pluripotency factors Nanog and Sox2 and indirectly Oct4 in combination with miR-296 and miR-470 (Tay et al., 2008; Niu et al., 2013). On the other hand, depending on the stage of the neuronal differentiation process, miR-134 has different targets and effects. For example, overexpression of miR-134 in cultures of E13.5 cortical NPs enhances their proliferation and counteracts apoptosis induced by Chordin-like 1 (Chrdl-1) and neuronal differentiation promoted by double-cortin (Dcx) through negatively regulating Chrdl-1 and Dcx expression (Gaughwin et al., 2011). In contrast, miR-134 reduces neuronal migration *in vitro* and *in vivo* in a Dcx-dependent manner (Schratt et al., 2006; Gaughwin et al., 2011). Thus, it would be very important to determine the molecular mechanisms regulating miR-134 expression and additional targets of this miRNA during the differentiation process of distinct types of neurons. Moreover, considering that miR-134 is not present in model organisms commonly used to study the neurogenic process such as *D. melanogaster* and zebrafish, a general role of this miRNA in the CNS development of different organisms is discarded, nonetheless its conservation in mammals suggests a critical role in the CNS development of more complex organisms.

Additionally, other members of the miR-379-410 cluster have been shown to regulate cell proliferation in the developing CNS. In this scenario, *in vivo* overexpression of miR-369-3p, miR-496 and miR-543 in radial glial cells (RGCs) which can differentiate into neurons, negatively regulate N-cadherin (Ncad) and lower levels of Ncad conduce to their premature neuronal differentiation which is prevented by expressing a miRNA-resistant Ncad version (Rago et al., 2014). On the other hand, when miR-369-3p, miR-496 and miR-543 are suppressed, an increase in cell proliferation is observed, which correlates with a decrease in neuronal differentiation (Rago et al., 2014). Moreover, in the same study it was shown that these three miRNAs not only control cell cycle and differentiation but also regulate migration of newborn neurons by negatively regulating the same target gene (Ncad) (Rago et al., 2014). Therefore, when these three miRNAs are overexpressed in immature neurons a delayed migration is observed and when the miRNAs are abrogated, new neurons are able to migrate within the cortical plate (Rago et al., 2014). These results are in agreement with a previous study showing that Ncad regulates neuron migration in the developing neocortex by mediating the interaction between the fibers of the RGCs and the migrating neurons (Shikanai et al., 2011). However, an important aspect of this study is the fact that these trio of miRNAs do not function as an on-off switch to regulate Ncad levels, but fine-tune its levels to control cell proliferation and neuronal differentiation in the neocortex. This fine-tuning mechanism could be used by other set of miRNAs as an important strategy to maintain critical protein levels to allow cell type-specific functions in one biological process. Thus, it would be interesting to study this kind of regulation to have a better understanding of the developing CNS.

### miR-137

Recent studies have demonstrated the expression of miR-137 in adult NSCs (Bier et al., 2013) as well as in different regions of the adult mouse brain including the amygdala, the hippocampus, the cerebral cortex and the hypothalamus (Herzer et al., 2012; Sun et al., 2012). Although it is known that a reduction of miR-137 is necessary for neuronal maturation and that by targeting the Mind bomb one (Mib1) ubiquitin ligase, miR-137 regulates processes such as dendritic morphogenesis, phenotypic maturation and spine development both in brain and cultured primary neurons (Szulwach et al., 2010; Smrt et al., 2011), a precise role for this miRNA during the early stages of neuronal differentiation during embryonic development has been not yet clearly identified. The orphan nuclear receptor TLX is expressed exclusively in the vertebrate forebrain and in embryonic brain; it is particularly expressed in the ventricular NSCs (Li et al., 2008). TLX positively regulates cell proliferation and self-renewal of mouse NSCs through activating the Wnt/β-catenin signaling pathway (Qu et al., 2010) and by inhibiting the cell cycle inhibitor *p21* and the tumor suppressor gene *pten* in embryonic brains (Li et al., 2008). Accordingly, in TLX^−/−^ embryonic forebrains, the negative regulation of *p21* and *pten* is lost resulting in reduced cell cycle progression of NSCs both *in vitro* and *in vivo* (Li et al., 2008). Recently it has been shown that miR-137 targets the histone lysine-specific demethylase 1 (LSD1) which is a transcriptional repressor of TLX (also known as Nr2e1) (Sun et al., 2012). Thus, miR-137 promotes TLX expression and NSCs self-renewal. In agreement with this, miR-137 overexpression promotes NSCs proliferation *in vitro* and *in vivo* (Szulwach et al., 2010). In contrast, after growth factor withdrawal miR-137 levels increase and promote the differentiation of NSCs cultures from adult mouse by targeting an entire different set of genes (Silber et al., 2008). Several studies have identified *Cdc42* and *Cdk6* as direct miR-137 target genes and their post-transcriptional silencing is associated with the induction of G1 cell cycle arrest resulting in neuronal differentiation of NSCs (Silber et al., 2008) and decreased cell growth and/or proliferation in different cellular contexts such as glioblastoma and colorectal carcinoma cells (Silber et al., 2008; Balaguer et al., 2011; Chen et al., 2011c,b; Liu et al., 2011a). In addition, miR-137 targets the histone demethylase Lysine (K)-Specific Demethylase 5B (Jarid1b or Jumonji), involved in the maintenance of the undifferentiated state of ESCs. When an anti-miR-137 is used, Jarid1b is not post-transcriptionally silenced and the differentiation of ESCs is blocked (Tarantino et al., 2010). Furthermore, miR-137 overexpression in neuroblastoma cell lines and in glioblastoma-derived cancer stem cells (GSCs) reduces cell viability and proliferation while, promoting neuronal differentiation (Althoff et al., 2013; Bier et al., 2013). Therefore, miR-137 may directly or indirectly regulate the expression of other “undifferentiated state” genes in the context of NSCs and NPs in order to preserve a proper CNS development. Consequently, miR-137 expression must be highly regulated to maintain the correct proliferative rate without losing the differentiation potential of these cells. In this sense, it is known that high levels of miR-137 promote NSCs proliferation and inhibit their differentiation, whereas decreased miR-137 expression promotes NSCs differentiation. Therefore, miR-137 expression must be tightly regulated; accordingly, it has been demonstrated that the DNA methyl-CpG-binding protein (MeCP2) and the stem cells specific TF, Sox2, negatively co-regulate miR-137 expression by decreasing the levels of the active chromatin-associated marks trimethyl histone H3 lysine 4 (H3-K4-Tri-Me) and acetylated histone H3 lysine 9 (H3-K9-Ac) and thus, inhibiting miR-137 transcription. Moreover, miR-137 targets the Ezh2 histone methyltransferase and Polycomb group (PcG) protein and by this, miR-137 feeds back to chromatin and results in a global decrease in the histone H3 trimethyl lysine 27 (H3-K27-Tri-Me) mark which contribute to inhibit miR-137 transcription and thus, to the modulation of the proliferation and differentiation of NSCs (Szulwach et al., 2010). Interestingly, miR-137 forms a regulatory feedback loop with TLX and LSD1 in which the regulator of NSCs self-renewal TLX represses the expression of miR-137 by recruiting LSD1 to the miR-137 genomic locus thus controlling the dynamics between the proliferative potential of NSCs and their differentiation during CNS development (Sun et al., 2012). It is clear that miR-137 expression should be tightly regulated in NSCs and NPs to maintain their undifferentiated and proliferative but still committed state during the embryonic development of the CNS. However, as miR-137 is conserved from *D. melanogaster* to vertebrates, future studies to identify new regulators and target genes of miR-137 will provide a better understanding of the mechanisms regulated by this microRNA during neuronal differentiation.

### miR-25

miR-25 forms part of the evolutionary conserved miR-106-25 cluster (Tanzer and Stadler, 2004) which is located within the thirteenth intron of the protein-coding gene *Mcm7*, a member of a DNA helicase family required for DNA replication. The miR-106-25 cluster has been reported to have proliferative and anti-apoptotic promoting effects (Kan et al., 2010). However, little is known about miR-25 functions during CNS development. miR-25 overexpression but not that of miR-106b or miR-93 promotes the proliferation of cultured NSCs and NPs from adult mice (Brett et al., 2011). This effect may in part results from direct regulation of the cell cycle inhibitor p57, a *bonafide* miR-25 target gene (Kim et al., 2009). Accordingly, in the developing spinal cord of the zebrafish embryo, Scratch2 prevents cell cycle re-entry of newly generated neurons by inhibiting miR-25 expression and therefore up-regulation of p57 expression (Rodríguez-Aznar et al., 2013). Likewise, the mechanisms that regulate miR-106-25 cluster expression in NSCs starts to be elucidated. Binding of the FoxO3 TF to the first intron of the *Mcm7* gene positively regulates the transcription of the miR-106-25/Mcm7 locus (Brett et al., 2011). However, it is unclear whether FoxO3 directly activates the transcription of this locus or indirectly inhibits the expression of a positive trans-acting factor that binds to the promoter of the miR-106-25 cluster (Renault et al., 2009). Nonetheless, the regulation of the miR-106-25 cluster by FoxO3 is crucial to induce the expression of genes involved in the maintenance of quiescence, in the prevention of premature neural differentiation as well as in the control of oxygen metabolism of NSCs. In accordance with this, bioinformatic predictions suggest genes involved in the p53-, hypoxia-, TGFβ-, insulin/IGF- and nitric oxide-signaling as promising putative target genes for miR-25 (Renault et al., 2009; Brett et al., 2011). However, further experiments are required to validate these predictions and the physiological relevance of these interactions.

### miRNAs as NP lineage regulators

**Figure 2:**
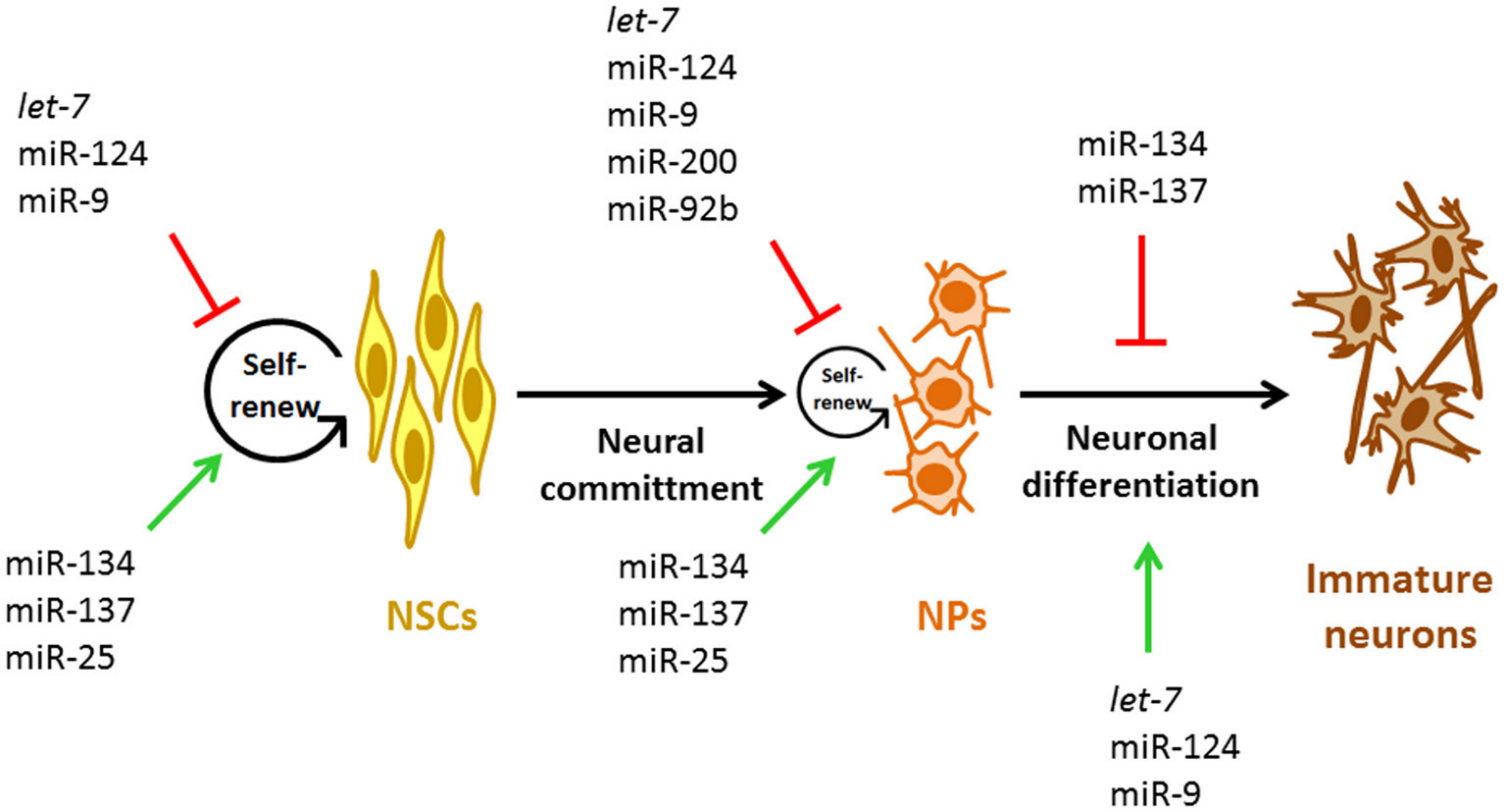
**miRNAs involved during early neurogenesis**. Representative miRNAs involved in the control of self-renewal and proliferation of NSCs and NPs and in the early stage neurogenesis. NSCs, neural stem cells; NPs, neural progenitors.

In addition to the mentioned functions of miRNAs on the NSCs biology, new experimental evidences point out the importance of miRNAs in the proliferative potential of NPs specifically (Figure 2, Table 1). miR-200 negatively regulates the expression of Sox2 and E2F3, a pluripotency factor and a cell cycle regulator, respectively (Johnson and Walker, 1999; Peng et al., 2012). The lack of Sox2 and E2F3 regulation by miR-200 results in reduced cell cycle exit and neuronal differentiation of ventral midbrain/hindbrain (vMH) NPs while, overexpression of miR-200 in primary vMH NPs results in the opposite effect (Peng et al., 2012) indicating that these interactions control the proliferative state of vMH NPs (Figure 2). Interestingly, both TFs Sox2 and E2F3 activate miR-200 transcription which establish a negative feedback loop between miR-200 and its target genes that guaranty NPs cell cycle exit and differentiation in the midbrain/hindbrain region (MHR) (Peng et al., 2012). Thus, it is possible that this kind of feedback loops between miRNAs and their target genes involved in cell cycle regulation represent a general mechanism to control the transition from pluripotent and multipotent cells such as NPs to post-mitotic cells in the developing CNS.

**Table 1:** **miRNAs involved during early neuronal differentiation**.

**miRNA**	**Target mRNA (organism)**	**Cell type**	**Biological effect**	**References**
*let-7a*	Lin28 (mouse)	NSCs	Neuronal lineage commitment	Rybak et al., 2008
*let-7b*	Cyclin D1 and TLX (mouse)	NSCs	Induction of neuronal differentiation	Zhao et al., 2010
*let-7d*	TLX (mouse)	NSCs	Inhibition of cell proliferation and induction of neuronal differentiation and migration	Zhao et al., 2013
miR-124	Jag1 (mouse)	NPs	Cell cycle exit and induction of neuronal differentiation	Liu et al., 2011b
miR-124	Sox9 (mouse)	NPs	Induction of neuronal differentiation	Cheng et al., 2009
miR-124	Scp1 (mouse)	NPs	Induction of neuronal differentiation	Visvanathan et al., 2007
miR-124	Ptbp1 (mouse)	NPs	Repression of alternative splicing of neuronal genes in non-neuronal tissues	Makeyev et al., 2007
miR-9	TLX (mouse)	NSCs	Reduction of cell proliferation and induction of neuronal differentiation	Zhao et al., 2009
miR-9	Stmn1 (mouse)	NPs	Increase in microtubule formation	Delaloy et al., 2010
miR-9	Hairy1 (frog)	Forebrain NPs	Inhibition of cell proliferation and induction of neuronal differentiation	Bonev et al., 2011
miR-9	Hairy1 (frog)	Hindbrain NPs	Inhibition of cell proliferation	Bonev et al., 2011
miR-9	Her5 and Her9 (zebrafish)	NSCs to NPs	Inhibition of cell proliferation and establishment of the midbrain-hindbrain boundary	Leucht et al., 2008
miR-9	FoxP1 (chicken)	Motor neuron subtypes	Motor neuron specification and columnar formation	Otaegi et al., 2011
miR-134	Nanog and Sox2 (mouse)	ESCs	Induction of differentiation into ectodermal lineages	Tay et al., 2008
miR-134	Nanog (mouse)	ESCs	Reduction of the self-renewal potential	Niu et al., 2013
miR-134	Chrdl-1 (mouse)	NPs	Inhibition of apoptosis and promotion of cell survival	Gaughwin et al., 2011
miR-134	Dcx (mouse)	NPs	Inhibition of neurogenesis	Gaughwin et al., 2011
miR-137	Jarid1b (mouse)	ESCs	Induction of cell differentiation	Tarantino et al., 2010
miR-137	Cdc42 and Cdk6 (mouse)	NSCs to NPs	Induction of G1 cell cycle arrest and induction of neuronal differentiation	Silber et al., 2008
miR-137	Ezh2 (mouse)	NSCs	Induction of cell proliferation	Szulwach et al., 2010
miR-25	Unknown	NSCs and NPs	Induction of cell proliferation	Brett et al., 2011
miR-25	p57 (zebrafish)	Immature neurons	Re-entry to cell cycle	Kim et al., 2009; Rodríguez-Aznar et al., 2013

*Lin28, Lin-28 homolog A; TLX, homolog of the Drosophila tailless gene; Jag1, Jagged1; Sox, SRY (sex determining region Y)-box; Scp1, CTD (carboxy-terminal domain RNA polymerase II polypeptide A) small phosphatase 1; Ptbp1, polypyrimidine tract binding protein 1; Stmn1, stathmin 1; Her, Hairy/E(spl) transcription factor; FoxP1, Forkhead box protein P1; Chrdl-1, Chordin-like 1; Dcx, double-cortin; Jarid1b or Jumonji, Lysine (K)-Specific Demethylase 5B; Cdc42, cell division control protein 42 homolog; Cdk6, cyclin-dependent kinase 6; Ezh2, Histone-lysine N-methyltransferase EZH2; NSCs, neural stem cells; NPs, neural progenitors; ESCs, embryonic stem cells*.

In the murine cortex, neurons arise from radial glia (direct neurogenesis) and also from intermediate NPs (indirect neurogenesis). In this sense, the generation of intermediate NPs is regulated by the TF Tbr2. Tbr2 is a marker of this class of cells. Moreover, the proliferative capacity of NPs is positively regulated by Tbr2 (Jan et al., 2013). The first evidence indicating that Tbr2 function could be controlled by miRNAs came from experiments where blocking the generation of mature miRNAs in murine cortical NPs resulted in increased numbers of Tbr2-expressing cells (Jan et al., 2013). Accordingly, miR-92b gain-of-function resulted in a rapid reduction of Tbr2-expressing cells and proliferating intermediate NPs (Jan et al., 2013); in contrast, specific miR-92b loss-of-function had opposite effects (Jan et al., 2013). These data strongly suggest that miR-92b limits the production and proliferation of intermediate cortical NPs (Figure 2) by negatively regulating Trb2 expression, which promote a tightly regulated neuronal output from radial glia and intermediate NPs by maintaining the balance between the intermediate NP and post-mitotic cell states.

miRNAs can also participate in specifying the identity of distinct NPs populations in different regions of the developing CNS (Table 1). In the ventral spinal cord there are five progenitor domains (p0-p2, pMN, and p3) that give rise to different neuronal populations such as ventral spinal interneurons or motor neurons (Chen et al., 2011a). These five progenitor domains are defined by specific TFs whose combinatorial expression must be tightly regulated in order to ensure the unequivocal assignment of NP identity (Chen et al., 2011a). Particularly, progenitors of spinal motor neurons (pMN) are specified by the TF Olig2, while V2 interneurons (p2) are specified by the TF Irx3 (Chen et al., 2011a). Although from the beginning of the spinal cord development the p2 progenitors express the pMN marker, Olig2, it is repressed by miR-17-3p through development progression, thus ensuring the proper specification of the pMN/p2 boundary and the production of V2 interneurons (Chen et al., 2011a). In this sense, mice lacking the miR-17/92 cluster present a dorsal shift in pMN/p2 boundary and incorrect production of V2 interneurons (Chen et al., 2011a). Therefore, Olig2 repression mediated by miR-17-3p is crucial for the correct patterning of ventral spinal NPs domains and thus, it is possible that other miRNAs also participate in NPs specifications in different CNS regions.

## miRNAs as promoters of neuronal fate and neurogenesis

NSCs and NPs have a high self-renewal potential and thus, can differentiate in any type of CNS cell including neurons and glial cells. During CNS development, NSCs and NPs undergo through defined steps to become a specific neural cell type. The transition from one stage to the next depends on extracellular cues that control NSCs and NPs self-renewal, proliferation and the differentiation potential by regulating intracellular signaling cascades. Among the miRNAs with an essential role in neuronal differentiation are *let-7*, miR-124, and miR-9 (Figure 2, Table 1). Interestingly, these three miRNAs are highly conserved during evolution and information regarding their role in the commitment of NSCs and NPs for a neuronal fate is presented below.

### let-7

The *let-7* family of miRNAs consists of 7 members in rat, 8 members in human and 10 members in mouse and zebrafish and it is the miRNA family with the highest expression in NSCs and NPs (Åkerblom and Jakobsson, 2013). In particular, *let-7b*, was identified in the mammalian brain whose expression is increased during *in vitro* neural differentiation (Sempere et al., 2004). The role of *let-7b* during the CNS development was characterized by the negative regulation that it exerts over different target genes involved in cell cycle control such as Cyclin D1 and the nuclear receptor TLX in NSCs (Zhao et al., 2010). *let-7a* induces neuronal lineage commitment of cultured mouse NSCs by targeting *lin-28* which inhibits pre-let-7 processing by Dicer in ESCs and thus, contribute to the maintenance of the NSCs self-renewal capacity (Rybak et al., 2008). Accordingly, the inhibition of *let-7b*, another member of the *let-7* family, favors the proliferative potential of NSCs and blocks their neuronal differentiation potential (Zhao et al., 2010). Interestingly, downregulation of TLX can also be mediated by *let-7d*, another member of the *let-7* family. *let-7d* overexpression reduces NSCs proliferation and promotes premature neuronal differentiation and migration (Zhao et al., 2013). Despite several *let-7* target genes are known, further studies are required to determine the upstream signaling pathways regulating *let-7* expression. Likewise, additional studies are required to determine the specific molecular mechanisms controlled by *let-7* during CNS development.

### miR-124

miR-124 is one of the most enriched miRNAs within the CNS (Landgraf et al., 2007). Its mature sequence is conserved from nematodes to primates (Reviewed in Meza-Sosa et al., 2012). miR-124 is not expressed in NSCs and its expression begins during the transition from NSCs to NPs (Åkerblom and Jakobsson, 2013). miR-124 levels increase when P19 cells are induced to differentiate into neurons by RA treatment (Sempere et al., 2004; Åkerblom et al., 2012). Thus, miR-124 pro-neuronal functions have been mainly described during the terminal neuronal differentiation processes such as neurite outgrowth where it alters the subcellular localization and expression of different members of the Rho GTPase family (Yu et al., 2008). Considering that an essential step in neurogenesis is the irreversible cell cycle exit, miR-124 gain and loss of function experiments have been important in elucidating how miR-124 participates in early events of embryonic neuronal differentiation. Thus, overexpression of miR-124 in cultured NSCs and embryonic cortical NPs induced a neuronal phenotype (Maiorano and Mallamaci, 2009). In contrast, inhibiting miR-124 expression *in vitro*, prevented the commitment for a neuronal fate while the proliferation of NSCs was promoted (Cheng et al., 2009). This might involve regulation the Notch signaling, which via the binding of Notch to the Jagged1 (Jag1) receptor is required for the maintenance of the self-renewal capacity of cultured NSCs and NPs and for blocking their differentiation (Imayoshi and Kageyama, 2011). Interestingly, transfection of NPs with miR-124 results in cell cycle exit and neuronal differentiation due to the Jag1 negative regulation mediated by miR-124 and consequently the inactivation of Notch signals (Liu et al., 2011b). Together, these studies indicate that miR-124 expression is essential for the induction of a neuronal cell fate by inducing NSC exit the cell cycle (Makeyev et al., 2007; Visvanathan et al., 2007). Thus, miR-124 plays a key role to promote neuronal differentiation of NSCs and NPs and it acts as a neuronal fate determinant and not only in neuronal terminal differentiation.

Once cells are committed to acquire a neuronal phenotype, miR-124 promotes NPs differentiation by regulating an intricate network of CNS-specific alternative splicing, specifically miR-124 targets the Polypyrimidine tract-binding protein 1 (Ptbp1) that represses the alternative splicing of neuronal genes in non-neuronal tissues (Makeyev et al., 2007). In addition, miR-124 promotes the transition from NPs to mature neurons by inhibiting non-neuronal genes such as *scp1* and *sox9* (Visvanathan et al., 2007; Cheng et al., 2009). On the other hand, the transition of self-renewing NSCs and NPs to post-mitotic cells requires, a switch in the ATP-dependent chromatin remodeling complex in which the BAF53a subunit of the NPs-specific BAF complex (npBAF) is replaced by the BAF53b to form the neuron-specific BAF complex (nBAF) (Yoo et al., 2009). By co-expressing a reporter that consists of BAF53a with its complete 3′ UTR and miR-124 or miR-9^*^ in the mouse neural tube of embryonic (E) day 11.5 (E 11.5), it was demonstrated that miR-124 together with miR-9^*^ inhibits BAF53a expression allowing BAF53b to be expressed in post-mitotic cells which correlates with diminished proliferation and increased dendritic outgrowth of these cells (Yoo et al., 2009). In addition, point mutations in the binding sites for miR-124 and miR-9^*^ within the BAF53a 3′ UTR, resulted in increased cell proliferation of NPs and consequently their neuronal differentiation was inhibited (Yoo et al., 2009).

Although several targets of miR-124 are well documented, the molecular mechanisms that regulate miR-124 expression during the first stages of neuronal differentiation have been little explored. In an *in vitro* model of neuronal differentiation, RA induced the expression of 19 miRNAs, including miR-124 (Sempere et al., 2004). However, miR-124 expression was detected by day four after RA-treatment which corresponds to a second wave of transcriptional activation of protein coding genes involved in neuronal terminal differentiation (Sempere et al., 2004). Evidently, additional studies are required to define the signals that promote miR-124 expression during early neuronal differentiation.

As mentioned, miR-124 is highly conserved through diverse species. However, the targets and thus the mechanisms that miR-124 regulates, are different between these organisms. For example, inhibition of miR-124 in *Caenorhabditis elegans* (*C. elegans*) does not affect differentiation of sensory neurons (Clark et al., 2010). In contrast to the main expression of mouse miR-124 in mature neurons, in the fruit fly *Drosophila melanogaster* (*D. melanogaster*); miR-124 is normally expressed not only in differentiating post-mitotic neurons but also, in proliferating NPs. In this sense, when miR-124 is knocked-down in neuroblasts of the developing larval brain, the number of post-mitotic neurons is reduced; however the neuronal cell fates acquired by these NPs are not affected. The reduction in post-mitotic cells results from a decreased proliferative capacity of miR-124-deficient NPs due to *anachronism* (*ana*) gene up regulation, a miR-124 direct target, which normally negatively regulates cell proliferation (Weng and Cohen, 2012). Thus, in contrast to miR-124 positive role in mouse neuronal differentiation, in *D. melanogaster* miR-124 supports neuroblasts proliferation. Further studies are necessary to uncover the genetic and/or epigenetic mechanisms that regulate miR-124 processing and/or expression in the first stages of neuronal differentiation providing a better understanding of the molecular mechanisms that maintain a correct number of NSCs and NPs across the species in which this miR-124 is conserved.

### miR-9

miR-9 is also a brain-enriched miRNA (Landgraf et al., 2007) and it is evolutionary conserved from flies to human (Yuva-Aydemir et al., 2011). Ablation of miR-9 in mice causes defects in the production of Cajal-Retzius cells, resulting from premature birth of cortical neurons and suppression of NPs proliferation in the ventricular and subventricular zones (Shibata et al., 2011) pointing out an important role of this miRNA in the CNS development. Participation of miR-9 has been widely characterized during the different stages of neuronal differentiation including its role during the first stages of CNS embryonic development. It is known that when NPs exit from the cell cycle and become post-mitotic cells, they require a precise balance between their proliferative and migratory rates in order to complete a successful maturation process (Delaloy et al., 2010). Accordingly, miR-9 inhibits NPs migration by targeting stathmin (Stmn1), which normally facilitates migration by increasing microtubule instability. In this sense, miR-9 overexpression promotes the proliferation of NPs derived from human ESCs and at the same time, it inhibits their migratory capacity by targeting Stmn1 resulting in enhanced microtubule formation that causes a delay of NPs to progress to a more mature NP fate in which they need not only to exit cell cycle but also, to migrate (Delaloy et al., 2010). Moreover, in the absence of miR-9, NPs present high levels of Stmn1 and enhanced migration *in vitro*. Interestingly, these effects do not lead to NPs early differentiation, in part because of partial inhibition of Stmn1 prevents the effects of miR-9 loss on proliferation and migration and both cellular properties are needed to maintain a proper neurogenic process (Delaloy et al., 2010). Moreover, regulation mediated by miR-9 is necessary for the development of the forebrain and hindbrain in the frog CNS, as NPs that lack miR-9 in the forebrain undergo apoptosis while, hindbrain NPs that lack miR-9 cannot exit from the cell cycle resulting in an accumulation of NPs and therefore, in a diminished rate of neuronal differentiation (Bonev et al., 2011). These opposite effects of miR-9 between forebrain and hindbrain are explained by the fact that miR-9 regulate specific target genes in each brain region (Bonev et al., 2011). When miR-9 is inhibited in the neural tube of *Xenopus tropicalis* (*X. tropicalis*), its target gene *hairy1* is upregulated resulting in higher levels of the fibroblast growth factor (Fgf) 8 (Fgf8) which is known to promote cell proliferation in the developing forebrain and in lower levels of the p53 negative regulator, E3 ubiquitin-protein ligase (Mdm2) (Bonev et al., 2011). In the developing forebrain miR-9 prevents p53 activation and apoptosis by reducing proliferative signals and lowering Mdm2 protein levels. Mdm2 is negatively regulated by several miRNAs including miR-192 (Pichiorri et al., 2010), miR-194 (Pichiorri et al., 2010), miR-215 (Pichiorri et al., 2010), miR-221 (Kim et al., 2010), and miR-17 (Li and Yang, 2012) in different cellular contexts; however, whether these or other miRNAs regulate Mdm2 expression during the CNS development must be determined. In contrast, in the hindbrain *hairy1* up regulation, as a consequence of miR-9 inhibition, leads to Zic1 activation that results in Wnt1 induction. Thus, resulting in higher expression of Cyclin D1 and enhanced cell proliferation rate.

In accordance to its high degree of conservation, the zebrafish miR-9 also modulates the establishment of the midbrain-hindbrain boundary (MHB) and keeps the balance between maintenance and differentiation of NSCs and NPs by targeting various genes of the Fgf signaling including *fgf8-1* and *fgfr1* and the anti-neurogenic genes *her5* and *her9* (Leucht et al., 2008). By overexpressing and knocking-down miR-9, it has been demonstrated that in the developing chick spinal cord, miR-9 defines motor neurons in the lateral motor columns (LMC) and pre-ganglionic autonomic motor neurons termed Column of Terni (CT) neurons by targeting FoxP1 (Otaegi et al., 2011). Additionally, in the mouse, miR-9 participates in a feedback regulatory loop that involves TLX, which represses the transcription of miR-9 and miR-9 negatively regulates TLX’s mRNA and protein levels inhibiting NSCs proliferation and inducing their differentiation (Denli et al., 2009; Zhao et al., 2009). In proliferating mouse NSCs, TLX predominates in this loop since it is expressed at higher levels while in differentiated cells miR-9 is highly expressed and TLX expression is inhibited (Denli et al., 2009; Zhao et al., 2009). In addition, a recent report showed that miR-9 targets Cyclin D1 mRNA in a gastric cell line (Zheng et al., 2013). Thus, it is possible that besides regulating TLX expression, miR-9 controls the proliferation rate of NSCs and NPs by regulating Cyclin D1 protein levels. The mentioned studies identified miR-9 as a coordinator not only of the proliferation of NSCs and NPs but also of the migration of NPs during the development of different organisms. However, as miR-9 has distinct target genes depending on the temporality of the CNS development and even between different organisms, the study of the regulation of this miR-9 during CNS development would provide valuable information to better understand the different neural cells differentiation programs.

## Conclusion and perspectives

Neurogenesis requires a very finely regulated gene expression network including positive and negative signals from both the intra- and extra-cellular environments. Diverse studies have demonstrated the essential role of miRNAs during CNS development as these molecules are critical regulators of gene expression through all the stages of neurogenesis, from the maintenance of the pluripotent state of ESCs to the establishment of neural phenotypes. However, many aspects about the target genes that a single miRNA regulates during the neurogenic process within different organisms and the molecular mechanisms that control miRNAs biogenesis, expression, and/or function are still uncovered. Furthermore, the fact that a single mRNA can be targeted by multiple miRNAs must be also considered.

The generation of specific neuronal phenotypes resides on the expression and function of specific TFs that activate or repress the transcription of their target genes in specific windows of time and space (Diez del Corral and Storey, 2001). There is evidence showing that a single TF or combination of certain TFs induces NSCs and/or NPs to differentiate into specific types of neurons within different regions of the mammalian brain as reported for Pax6 for differentiation of dopaminergic neurons in the olfactory bulb (Kohwi et al., 2005), neurogenin 3 (Ngn3) for POMC, NPY, TH, and SF1 neurons in the hypothalamus (Pelling et al., 2011), LIM homeobox 6 (Lhx6) for GABAergic interneurons and somatostatin (Sst) interneurons in the cortex (Neves et al., 2013) and LIM homeobox 7 (Lhx7) for cholinergic interneurons in the stratium (Lopes et al., 2012). Although considerable efforts have been made to identify the TFs that determine neuronal subtypes, the molecular mechanisms controlling their expression are beginning to be elucidated. In this sense, post-transcriptional regulation mediated by miRNAs during neuronal cell fate determination and neurogenesis stages, has a key role (Ivey and Srivastava, 2010; Li and Jin, 2010). Interestingly, the concentration gradient of a single miRNA is capable of determining specific zones of neuronal differentiation as it is the case of miR-7 that maintains the proper localization of dopaminergic neuronal differentiation regions within the mouse olfactory bulb by having an opposite concentration/expression gradient to that of its target gene, Pax6 (De Chevigny et al., 2012). Thus, it is possible that this case is not unique and different circuits of miRNAs and target mRNAs function as neuronal cell fate determinants in other regions of the brain. This might also be the case in invertebrates organisms such as *D. melanogaster* and *C. elegans*, since most of the miRNAs with a role during developmental programs are highly evolutionary conserved (Rajasethupathy et al., 2009; Yuva-Aydemir et al., 2011). Given that both TFs and miRNAs are expressed in specific windows of time and space at different stages of neuronal differentiation and that miRNAs have more than one target gene, genome wide approaches focused in identifying the expression pattern of miRNAs and their target genes during specific times and brain regions during the CNS development of different model organisms, would provide valuable information. This would give us a more complete scenario and better understanding of the functions of these miRNA:mRNA circuits. Additionally, the use of new high throughput techniques such as crosslinking, ligation, and sequencing of hybrids (CLASH) has pointed out that miRNAs do not only recognize perfect complementary binding sites at the 3′ UTR but also, within the 5′ UTR (Tsai et al., 2009; Helwak et al., 2013), the open reading frame (ORF) (Faghihi et al., 2010; Helwak et al., 2013) or with seedless binding at the 3′ UTR of the target mRNA (Lal et al., 2009; Helwak et al., 2013). In this sense, the identification of new rules of base pairing between miRNAs and their target genes has opened a new point of view for miRNA-mediated gene regulation in part because the efficiency of the different targeting strategies may affect the functionality of the RISC and therefore the cellular output in a defined moment. In other words, it would be possible that different cellular responses may be obtained due to the dynamic miRNA:mRNA interactions that can co-exist in one biological process. Thus, this new layer of regulation should be considered when the complexity of the gene regulation required for neurogenesis is analyzed.

Despite the vast quantity of studies focused on the identification of miRNAs target genes and their effects in CNS development, the regulation of the biogenesis, expression, and/or function of these post-transcriptional regulators has been scarcely explored. A recent report, demonstrated that the processing of the miR-7 pre-miRNA generated from the heterogeneous nuclear ribonucleoprotein K (hnRNP K) pre-mRNA transcript, is inhibited in non-brain human and mouse cells due to the binding of the RNA binding proteins (RBPs) Musashi homolog 2 (MSI2) and Hu antigen R (HuR) to the terminal loop of the pri-miR-7 and the stabilization of the pri-miRNA structure (Choudhury et al., 2013). Moreover, knock-out mice for MSI2 present higher levels of mature miR-7 without a change in pri-miR-7 abundance confirming that RBPs are key players in the regulatory mechanism controlling miRNA biogenesis (Choudhury et al., 2013). In addition, miR-7 biogenesis regulation also occurs *via* MSI2 and HuR binding during the *in vitro* neuronal differentiation of the SH-SY5Y cell line (Choudhury et al., 2013). Another study showed that the control of miR-7 biogenesis by the quaking (QKI) RBPs, isoforms QKI-5 and QKI-6 that are localized at the nucleus and throughout the cell respectively, contribute to regulate the proliferation rate of glioblastoma cells cultures (Wang et al., 2013). Absence of QKI-5 and QKI-6 results in increased mature miR-7 levels due to the fact that these proteins negatively regulate pri-miR-7 to miR-7 processing by maintaining the pri-miR-7 at the nucleus and tightly bounded by Drosha (Wang et al., 2013). Silencing QKI in the U343 glioblastoma cell line, results in miR-7 expression and cell cycle arrest, through a mechanism involving miR-7 negative regulation of epidermal growth factor (EGF) receptor (EGFR) protein levels, thus blunting the EGF-dependent ERK activation (Wang et al., 2013). Therefore, it is possible that other brain-enriched miRNAs such as miR-124, miR-9 and *let-7* and even, other tissue-specific miRNAs could be regulated at their biogenesis, depending on the intra- and extra-cellular conditions that define the different developmental stages of a given cellular differentiation program. Moreover, the participation of defined tissue-specific factors as RBPs cannot be discarded and may provide an additional layer of regulation.

The activity of miRNAs can also be regulated. In this sense, another class of ncRNAs known as long-ncRNAs including natural antisense transcripts and circular RNAs (circRNAs) has been reported to regulate miRNA function primarily by sequestering their mature forms through mimicking their original target genes or by competition with other regulatory RNAs for binding to their target mRNAs (Faghihi et al., 2010; Hansen et al., 2013; Memczak et al., 2013). At least, 1000 pairs of natural sense-antisense transcripts are well conserved between the human and the mouse genomes (Faghihi et al., 2010) and one of them, the β-secretase 1 (BACE1) antisense transcript, which is upregulated in the brain of Alzheimer disease (AD) patients, promotes the stability of the BACE1 sense transcript (Faghihi et al., 2010). Recently, it was shown that the BACE1 antisense transcript competes with miR-485 for binding within the ORF of the BACE1 mRNA. When the natural antisense BACE1 transcript binds to the BACE1 mRNA, it blocks the negative effect of miR-485 (Faghihi et al., 2010). Accordingly, in AD patients, the expression patterns of BACE1 antisense transcript and miR-485 are deregulated compared to healthy individuals (Faghihi et al., 2010). On the other hand, recent studies revealed a regulatory role for circRNAs by functioning as natural miRNA sponges and regulating the CNS development (Hansen et al., 2013; Memczak et al., 2013). The first genome-wide study demonstrated the expression of thousands of stable circRNAs in the human, mouse and nematode genomes most of which showed tissue or developmental stage specific expression (Memczak et al., 2013). In particular, the human circRNA antisense to the cerebellar degeneration-related protein 1 transcript (CDR1as) contains 63 conserved binding sites for miR-7 and specifically regulates miR-7 expression in neuronal tissues (Memczak et al., 2013). Moreover, due to the high degree of conservation of miR-7, the binding sites in the human CDR1as are functional when it is expressed in zebrafish resulting in impaired midbrain development which is similar to the phenotype of knocking-down miR-7 (Memczak et al., 2013). An independent study, described ciRS-7, another circRNA, as a miR-7 sponge in the human brain and in mouse neocortical and hippocampal neurons (Hansen et al., 2013). ciRS-7 contains more than 70 conserved binding sites for miR-7 and when miR-7 binds to it, AGO is recruited and binds to ciRS-7:miR-7 complexes however, ciRS-7 is resistant to miR-7-mediated destabilization resulting in miR-7 activity blockage and derepression of miR-7 target genes (Hansen et al., 2013). An interesting finding is the miR-671-directed cleavage of ciRS-7 due to the perfect sequence complementarity that exists between both RNAs (Hansen et al., 2013). This observation suggest that miR-671 might function as an indirect regulator of miR-7 activity by targeting and reducing ciRS-7 levels; however, the exact function of the ciRS-7:miR-671 interaction during the development of the CNS is still unknown.

Thus, circRNAs can regulate miRNA activity within the CNS and therefore adds a new layer of regulation that may provide major specificity and fine-tuning of gene expression during the different stages of neurogenesis that are crucial for the proper functioning of neurons generated from NSCs and NPs. In addition, it is possible that this phenomenon is not particular of the CNS as a testis-specific circRNA serves as a miR-138 sponge (Hansen et al., 2013). Thus, regulation by long-ncRNAs seems to be important for controlling miRNA expression and activity in the CNS. However, more functional analysis of naturally expressed circRNAs within the CNS will provide useful information regarding the precise role of circRNAs and other long-ncRNAs in the regulation of gene expression required through the different developmental stages of the CNS and also, whether miRNAs other than miR-7 are regulated by long-ncRNAs. As remarked, there is an interface in which distinct groups of regulatory RNAs interact together with defined tissue and/or time/space-specific factors to control the expression of different mRNAs and give the necessary output of gene expression and protein synthesis depending on the cellular context and/or intra- and extra-cellular conditions. Therefore, the identification of new players within these intricate regulatory gene expression networks and the definition of their role during normal or pathological conditions of the CNS would provide a better understanding of the biological processes and times in which miRNAs, their regulators, and their target genes act. For this, genome-wide as well as system biology approaches represent promising tools for the development of new diagnostic and therapeutic strategies for the prevention or treatment of CNS developmental disorders in which the differentiation and/or function of specific types of neurons is compromised.

In conclusion, it is important to highlight that although several miRNAs have been identified as key molecules for the progression of the different stages of CNS development, their role in the specification of neuron subtypes and the molecular mechanisms that dictate their expression during the neurogenic process remain largely unknown. Moreover, miRNA role in regulating the function and specific properties of NSCs and NPs and their irreversible exit from the cell cycle has been little explored. In light of the fact that miRNAs have very precise expression patterns depending on the cell type, tissue and/or developmental stage; it is challenging to generalize a single mechanism to regulate their expression and to identify the target genes that each miRNA has during each stage of neurogenesis. Thus, the combination of bioinformatic tools and experimental techniques will help in the study of miRNAs role in early neurogenesis and how they, their target genes, and their regulators are integrated within the regulatory gene expression networks that determine each particular neuronal phenotype.

### Conflict of interest statement

The authors declare that the research was conducted in the absence of any commercial or financial relationships that could be construed as a potential conflict of interest.
